# The association between unintended pregnancy and maternal mental health in rural China

**DOI:** 10.3389/fpubh.2025.1498473

**Published:** 2025-03-07

**Authors:** Nan Wang, Yunjie Liu, Jianmin Ai, Jingchun Nie, Jie Yang

**Affiliations:** Center for Experimental Economics in Education, Shaanxi Normal University, Xi’an, China

**Keywords:** unintended pregnancy, depression, anxiety, stress, maternal women, rural China

## Abstract

**Background:**

Unintended pregnancies are common in rural China. However, the association between unintended pregnancy and maternal mental health remains poorly understood. This study aimed to assess the prevalence of unintended pregnancies, their association with mental health concerns, and the contributing factors in rural China.

**Methods:**

A cross-sectional design was used in this study. We included 473 pregnant women (age ranging from 18 to 45 years; local residency for at least 1 year; current pregnancy) from 10 counties in rural areas of Shaanxi Province, which is highly representative of rural northwest China in terms of economic status, geographical characteristics, and traditional culture. Mental health was assessed using the Depression, Anxiety, and Stress Scales-21 (DASS-21), widely recognized for its reliability, validity, and applicability in the Chinese context. Descriptive statistics and logistic regression analyses were employed to elucidate the prevalence of unintended pregnancy issues and explored their association with maternal mental health.

**Results:**

The prevalence rates of depression, anxiety, and stress in the full sample were 19.24, 23.68, and 10.99%, respectively. The proportion of unintended pregnancies was 41.44%, with significantly higher rates of depression, anxiety, and stress tendencies compared to intended pregnancy. Logistic regression analysis revealed a significant association between unintended pregnancy and an increased risk of anxiety tendency (OR = 1.96, 95% CI = 1.25–3.08, *p* = 0.004) as well as stress tendency (OR = 2.15, 95% CI = 1.15–4.02, *p* = 0.017). Furthermore, among women with unintended pregnancy, anxiety tendency was more pronounced among unemployed women (OR = 2.05, 95% CI = 1.25–3.35, *p* = 0.004), and co-residing with their mother-in-law (OR = 2.47, 95% CI = 1.40–4.38, *p* = 0.002). Similarly, stress tendency was more pronounced among unemployed women (OR = 2.20, 95% CI = 1.11–4.34, *p* = 0.023), and co-residing with their mother-in-law (OR = 2.60, 95% CI = 1.17–5.74, *p* = 0.018).

**Conclusion:**

The positive correlation exists between unintended pregnancy and maternal mental health risks. The high prevalence of unintended pregnancies underscores the need for policies aimed at reducing their occurrence, as well as interventions targeting mental health support to pregnant women.

## 1 Background

Unintended pregnancies (UIP), defined as pregnancies that are either mistimed or unwanted at the time of conception, remain a significant issue globally (1, 2). Unintended pregnancy poses a significant global public health challenge, as evidenced by World Health Organization (WHO) indicating that 74 million women in low- and middle-income countries experience unintended pregnancies annually (3). Research conducted in China reveals that 42.4% of married Chinese women within the reproductive age group experience unintended pregnancies (4), Chinese traditional culture hinders public discussions on sex-related issues, including contraception methods. Consequently, limited knowledge about preventing unintended pregnancies and restricted access to contraceptives persist due to economic and cultural factors (4–6). In major urban areas of China, the prevalence of unintended pregnancies among women in their reproductive years remains persistently high, reaching nearly 30 percent (7). The proportion of unintended pregnancies is likely to be higher in rural areas of China, primarily due to a lack of knowledge regarding contraception (8–10). Therefore, the issue of unintended pregnancy warrants attention.

Maternal mental health is one of the significant global concerns. Meta-analyses show that perinatal depression affects 10–15% of women in developed countries, with postnatal depression impacting about 10–12% of mothers (11). A Canadian study of 615 mothers identified three anxiety trajectories: very low and stable (13%), moderate and stable (29%) (12). Research also indicates that perceived stress during pregnancy ranges from 5.5 to 15% in developed countries and 33 to 52.9% in developing countries (13). Maternal depression is an increasingly serious issue in China. A meta-study in mainland China found that maternal depression prevalence was 16.3%, roughly 1.5–2.0 times higher than general population depression rates (14). A study from Shanghai in China showed that 11.07% of maternal women experienced depression, 5.42% anxiety, and 34.85% elevated stress levels (15). The severity of maternal depression can be even worse in rural areas of China. The rates of prenatal and postnatal depression in rural China were 19.5 and 18.6%, respectively (16).

Relevant factors influencing maternal depression have been extensively documented in previous literature. The factors encompass personal aspects such as demographic characteristics, knowledge, behaviors, and attitudes (17). Additionally, family-related elements including support from family members and dynamics within marital relationships and relationships with parents-in-law play a significant mediating role (18, 19). Moreover, social factors like the presence of extensive social networks have been shown to mitigate the likelihood of experiencing depressive symptoms (19, 20). These findings emphasize that becoming a mother is a unique experience for women, requiring extensive knowledge and preparation to navigate parenting challenges and adapt to their new lives.

The unintended pregnancy may pose a risk to maternal mental health, as it suggests that they may not be ready to assume parental responsibilities. Unintended pregnancy refers to a pregnancy that is unwanted (occurred when no children or no more children were desired) or mistimed (occurred earlier than desired). On one hand, an unintended pregnancy can disrupt future plans, such as work and living arrangements, and create uncertainty about handling the responsibilities of motherhood (21). On the other hand, it undoubtedly added to their financial burden due to significant additional expenses (22). Therefore, these challenges associated with unintended pregnancy can negatively impact their mental well-being. Although some research has explored the association between unintended pregnancies and maternal mental health during pregnancy, these studies have primarily focused on developed countries or urban areas in developing countries (22–24). Despite the high prevalence of unintended pregnancies in rural areas, there is a lack of research exploring their impact on maternal mental health in these settings.

Based on a population-based survey in the rural areas of northeastern China, this study aims to assess the prevalence of unintended pregnancies, their association with mental health concerns, and the contributing factors in rural China. Based on the research findings, this research can provide more targeted policy recommendations and guidance.

## 2 Methods

### 2.1 Setting

Our study was conducted in five prefectures located within the rural areas of Shaanxi Province, renowned for its diverse geography including both mountainous and plain regions. Among these prefectures, three were situated in mountainous areas while the remaining two were situated in plains. As of 2022, the population of Shaanxi Province stood at 39.6 million individuals, with 14.2 million residing in rural areas, accounting for approximately 36.0% of the total population. First, the per capita disposable income of rural residents in Shaanxi Province was $2,155 (25), which closely approximated the national average ($2,612), thus serving as a reliable indicator for the economic development level in rural China. Second, Shaanxi Province is renowned for its exceptional geographical diversity, encompassing a wide range of terrain types such as mountains, plateaus, and plains. As such, it serves as an exemplary representation of the diverse geography found in western China. Third, With a history spanning over 1,200 years and serving as the capital of ancient China for 13 dynasties, Shaanxi Province stands as an exemplary representative of China’s rich traditional culture, which plays a crucial role in addressing unintended pregnancies. Therefore, the sample areas selected in this study can thus be regarded as representative of rural areas in China.”

### 2.2 Sampling

This study used a cross-sectional design. We collected in March 2021 from households located in rural areas. We applied a multilevel cluster random sampling method in the following steps. Five prefectures in Shaanxi Province were selected, and two counties were randomly chosen from each prefecture, totaling 10 counties. Within these counties, all towns—excluding county seats—were included in the sampling frame. For counties with more than 10 townships, we randomly selected 10; for those with fewer than 10, all townships were included. A list of all pregnant women in each township was then obtained with the assistance of the local health bureau, as all pregnant women were required to register with the bureau in order to obtain permits for accessing public health services such as prenatal examinations. Additionally, towns with fewer than three eligible women were excluded. During the sampling process, we cross-validated it with primary healthcare providers responsible for pregnant women’s care management in each village. Finally, 10 pregnant women were randomly selected from each township, resulting in an initial sample of 527 participants.

To ensure the representativeness of the targeted population and to address ethical and practical considerations, inclusion criteria for this study were as follows: (1) Age ranging from 18 to 45 years, corresponding to the age range associated with high fertility levels and optimal physical functioning; (2) local residency for at least 1 year, which is crucial to minimize potential data loss resulting from participant relocation or mobility; and (3) current pregnancy. The sample size was estimated to achieve a sampling standard error of 0.02 with a 95% confidence interval ranging from 0.17 to 0.21 for a binomial variable of 0.19, as determined from our pilot study. The planned sample size was set at 444. A total of 527 maternal participants were selected as the study sample actually, the sample size thus fulfills the minimum requirement for a valid sample.

Among them, 54 participants were excluded due to missing or incomplete survey responses. Specifically, 29 participants did not complete the DASS-21 scale, while 25 participants failed to provide information regarding their family income, employment status, or other relevant details, resulting in missing variables. Ultimately, the analysis included 473 participants from 10 counties.

### 2.3 Data collection

The data were collected via face-to-face interviews conducted by trained survey enumerators between March and April 2021. Enumerators received comprehensive training on administering the survey instruments for each main component of the study, followed by a pilot study involving 20 participants to ensure research reliability and validity. Prior to the interviews, eligible participants were provided with a consent form containing detailed information about program objectives, procedures, potential risks and benefits, as well as a privacy statement. Each interview was conducted individually to minimize disruptions from other family members. The survey collected data on family characteristics, demographic characteristics of the pregnant women, as well as their mental health.

### 2.4 Measurements and variable setting

#### 2.4.1 Dependent variable

Maternal mental health: The primary outcome of interest in this study is maternal mental health. Participants were administered the Depression, Anxiety, and Stress Scales-21 (DASS-21), a validated tool developed by Lovibond (26). The DASS-21 is utilized to assess symptoms of depression, anxiety, and stress in individuals (27). Previous studies have consistently demonstrated the high reliability and validity of the DASS-21 on a global scale (28, 29). The internal consistency of the Chinese DASS-21 was robust, with Cronbach’s alpha coefficients ranging from 0.80 to 0.93 for the depression, anxiety, and stress subscales, respectively, which indicates high reliability and validity in measuring the constructs of interest (30, 31). At the same time, the Chinese DASS-21 has demonstrated varying degrees of applicability among different population subgroups, including adolescents, pregnant women, and older adults (32–34).

The DASS-21 scale comprises 21 items, divided into three factors: depression, anxiety, and stress, each subscale has 7 items, and each item is scored from 0 (did not apply to me at all over the last week) to 3 (applied to me very much or most of the time over the past week). Each subscale score goes from 0 to 21, and the final score of each item group (Depression, Anxiety, and Stress) needs to be multiplied by two (x2), with higher scores suggesting a greater likelihood of depression, anxiety, or stress tendencies in the previous week.

According to DASS-21 guidelines (35), the DASS-21 scores in this study were converted into dummy variables for each subscale to indicate any tendency. Specifically, a score of “1” was assigned if the depression score was greater than 9, the anxiety score was greater than 7, or the stress score was greater than 14, indicating a tendency toward depression, anxiety, or stress, respectively. Otherwise, a score of “0” is assigned.

#### 2.4.2 Independent variables

Unintended pregnancy. It refers to a pregnancy that is unwanted (occurred when no children or no more children were desired) or mistimed (occurred earlier than desired) (36, 37). In the survey, participants were asked, “Was this pregnancy planned?” The responses were categorized as 1 for “Yes” and 0 for “No” (2, 38), it is a dummy variable.

#### 2.4.3 Socio-demographic characteristics

Socio-demographic characteristics: We collected demographic information on maternal, husband, and family characteristics. Maternal and paternal characteristics include their age, education level, employment status, and health status. Family characteristics include whether the women are co-residents with their mother-in-law, household size, and annual family income. All variables are dummy variables.

### 2.5 Statistical analysis

The data were analyzed using STATA 18.0 software. Maternal data were categorized based on pregnancy intention, and sociodemographic variables were described in terms of frequency and percentage. Chi-square tests were conducted to compare these variables across different pregnancy intention statuses. Furthermore, chi-square tests were performed to compare the means of depression tendency, anxiety tendency, and stress tendency among unintended pregnancy categories. Multivariate logistic regression was employed to analyze the associations between unintended pregnancy and maternal mental health (depression tendency, anxiety tendency, and stress tendency). Additionally, subgroup logistic regression analyses were conducted to examine the heterogeneous effects of unintended pregnancy on mental health tendencies among different groups of women categorized by age, employment status, and co-residency with mother-in-law. Statistical significance was set at *p* < 0.05, and odds ratios (ORs) with 95% confidence intervals (CIs) were reported as results.

## 3 Results

### 3.1 Sociodemographic characteristics of participants by pregnancy intention

The sociodemographic characteristics and their univariate associations with pregnancy intention are detailed in Table 1. A total of 473 women participated in this study. 233 (49.26%) of the women were 29 years old or older. 313 (66.17%) of women had an education level of junior high school or below. 405 (85.62%) of the women were unemployed. 345 (72.94%) of the women reported their health status as good. 198 (41.86%) of the husbands had an education level of above junior high school. The vast majority of husbands were employed (420; 88.79%). 314 (66.38%) of women co-resided with the mother-in-law, while the rest did not (159; 33.62%). 382 (80.76%) of the women’s household size had seven or fewer members. 291 (61.52%) of the families had an annual income of less than 6,9,200 CNY. Additionally, there were no significant differences in most sociodemographic characteristics between women with unintended pregnancies and those with intended pregnancies.

**Table 1:** Sociodemographic characteristics of participants by pregnancy intention.

Variables	Full sample		Intended pregnancy		Unintended pregnancy		X^2^	*p*-value
	n (473)	%	n (277)	% (58.56)	n (196)	% (41.44)		
Women characteristics								
Women age								
<29	240	50.74	140	50.54	100	51.02	0.01	0.918
≥29	233	49.26	137	49.46	96	48.98		
Women education								
Junior high school or below	313	66.17	178	64.26	135	68.88	1.09	0.296
Above junior high school	160	33.83	99	35.74	61	31.12		
Women employment status								
Unemployed	405	85.62	237	85.56	168	85.71	0.00	0.962
Employed	68	14.38	40	14.44	28	14.29		
Health status								
Not Good	128	27.06	73	26.35	55	28.06	0.17	0.681
Good	345	72.94	204	73.65	141	71.94		
Husband characteristics								
Husband age								
<31	240	50.74	146	52.71	94	47.96	1.04	0.309
≥31	233	49.26	131	47.29	102	52.04		
Husband education								
Junior high school or below	275	58.14	154	55.60	121	61.73	1.78	0.182
Above junior high school	198	41.86	123	44.40	75	38.27		
Husband employment status								
Unemployed	53	11.21	31	11.19	22	11.22	0.00	0.990
Employed	420	88.79	246	88.81	174	88.78		
Family characteristics								
Co-resident with mother-in-law								
Yes	314	66.38	187	67.51	127	64.80	1.08	0.298
No	159	33.62	91	32.49	69	35.20		
Household size								
≤7	382	80.76	234	84.48	148	75.51	5.94	0.015*
>7	91	19.24	43	15.52	48	24.49		
Annual family income								
<6,9200CNY	291	61.52	167	60.29	124	63.27	0.43	0.512
≥6,9200CNY	182	38.48	110	39.71	72	36.73		

*p < 0.05.

Among the 473 women, 196 (41.44%) experienced unintended pregnancies. As presented in Table 1, there were no significant disparities observed between women with unintended pregnancies and those with intended pregnancies concerning age of 29 years or older, employment status as employed (14.29% vs. 14.44%), or attainment of above junior high education level by either themselves or their husbands (31.12 vs. 35.74; 38.27 vs. 44.40). However, within household characteristics, a significantly higher proportion of women with larger household sizes was observed among those experiencing unintended pregnancies compared to those with intended pregnancies (household size over 7 members: 24.49% vs. 15.52%; *p* = 0.015). There were no significant differences regarding co-residence with mother-in-law (yes: 64.80% vs. 67.51%) or annual family income (≥ 69,200 CNY: 36.73% vs. 39.71%).

### 3.2 Prevalence of mental health tendencies by pregnancy intention

The mental health tendencies were compared between groups with unintended and intended pregnancies, as presented in Table 2. The prevalence rates of depression, anxiety, and stress in the full sample were 19.24, 23.68, and 10.99%, respectively. A significantly higher proportion of women with unintended pregnancy exhibited anxiety tendencies compared to those with intended pregnancy (29.59% vs. 19.49%, *p* = 0.011). Furthermore, women with unintended pregnancies also demonstrated a significantly higher prevalence of stress tendencies compared to those with intended pregnancies (14.8% vs. 8.3%, *p* = 0.026). Additionally, although not statistically significant, women with unintended pregnancies exhibited slightly higher levels of depression tendencies compared to those with intended pregnancies (21.94% vs. 17.33%).

**Table 2:** Mental health tendencies and their univariate associations with pregnancy intention.

	Depression tendency		Anxiety tendency		Stress tendency	
	Yes	No	Yes	No	Yes	No
Full sample	91(19.24%)	382(80.76%)	112(23.68%)	361(76.32%)	52(10.99%)	421(89.01%)
Intended pregnancy	48(17.33%)	229(82.67%)	54(19.49%)	223(80.51%)	23(8.30%)	254(91.70%)
Unintended pregnancy	43(21.94%)	153(78.06%)	58(29.59%)	138(70.41%)	29(14.80%)	167(85.20%)
X^2^	1.57		6.48		4.94	
*p*-value	0.211		0.011*		0.026*	

*p < 0.05.

### 3.3 Associations between unintended pregnancy and maternal mental health tendencies

The relationship between unintended pregnancies and tendencies toward depression, anxiety, and stress is presented in Table 3. Our findings indicated that women with unintended pregnancies had a significantly higher likelihood of experiencing anxiety tendencies (OR = 1.96; 95% confidence interval [CI] = 1.25–3.08; *p* = 0.004) and stress tendencies (OR = 2.15; 95% CI = 1.15–4.02; *p* = 0.017) compared to those with intended pregnancies. Although not statistically significant, women with unintended pregnancies also exhibited a slightly elevated likelihood of depression tendencies (OR = 1.35; 95% CI = 0.82–2.21; *p* = 0 0.234).

**Table 3:** Multivariate logistic regression analysis of unintended pregnancy for maternal mental health tendency.

	Depression tendency		Anxiety tendency		Stress tendency	
	OR (95% CI)	*p*-value	OR (95% CI)	*p*-value	OR (95% CI)	*p*-value
Unintended pregnancy						
No	1	0.234	1	0.004**	1	0.017*
Yes	1.35 (0.82,2.21)		1.96 (1.25, 3.08)		2.15 (1.15, 4.02)	
Women characteristics						
Women age						
<29	1	0.927	1	0.500	1	0.182
≥29	0.97 (0.52, 1.81)		0.82 (0.46, 1.49)		0.58 (0.27, 1.29)	
Women education						
Junior high school or below	1	0.144	1	0.136	1	0.052
Above junior high school	0.65 (0.36, 1.16)		0.67 (0.39, 1.13)		0.46 (0.21, 1.01)	
Women employment status						
Unemployed	1	0.970	1	0.893	1	0.444
Employed	0.98 (0.47, 2.06)		1.05 (0.55, 2.01)		1.43 (0.57, 3.56)	
Health status						
Not Good	1	0.000***	1	0.064	1	0.001**
Good	0.39 (0.23, 0.66)		0.62 (0.38, 1.03)		0.34 (0.17, 0.65)	
Husband characteristics						
Husband age						
<31	1	0.498	1	0.767	1	0.253
≥31	1.25 (0.66, 2.35)		0.92 (0.51, 1.64)		1.59 (0.71, 3.54)	
Husband education						
Junior high school or below	1	0.948	1	0.682	1	0.849
Above junior high school	1.02 (0.60, 1.72)		1.11 (0.69, 1.78)		1.07 (0.55, 2.07)	
Husband employment status						
Unemployed	1	0.521	1	0.368	1	0.372
Employed	0.78 (0.37, 1.66)		0.73 (0.36, 1.46)		0.66 (0.26, 1.65)	
Family characteristics						
Co-resident with mother-in-law						
No	1	0.140	1	0.333	1	0.295
Yes	1.51 (0.87, 2.62)		1.28 (0.78, 2.10)		1.45 (0.72, 2.92)	
Household size						
≤7	1	0.173	1	0.017*	1	0.033*
>7	0.63 (0.31, 1.23)		0.46 (0.24, 0.87)		0.35 (0.14, 0.92)	
Annual family income						
<6.92	1	0.984	1	0.385	1	0.130
≥6.92	1.01 (0.59, 1.71)		1.24 (0.77, 1.99)		1.67 (0.86, 3.25)	

*p < 0.05, **p < 0.01.

### 3.4 Heterogeneous analysis

The association between unintended pregnancies and maternal mental health across groups with different characteristics is demonstrated in Table 4. Regarding age group, the relationship between unintended pregnancy and maternal mental health remained consistent regardless of whether the woman was younger or older than 29 years (OR = 1.36, 95% CI = 0.64–2.87 vs. OR = 1.60, 95% CI = 0.77–3.33 for depression tendency; OR = 2.08, 95% CI = 1.08–4.01 vs. OR = 2.18, 95% CI = 1.09–4.34 for anxiety tendency; OR = 2.34, 95% CI = 0.95–5.74 vs. OR = 2.45, 95% CI = 0.92–6.51 for stress tendency). The association between unintended pregnancy and maternal mental health was found to be stronger among employed women compared to unemployed women (OR = 2.81, 95% CI = 0.34–23.06 vs. OR = 1.24, 95% CI = 0.72–2.12 for depression tendency; OR = 8.99, 95% CI = 0.33–24.51 vs. OR = 2.20, 95% CI = 1.11–4.34 for stress tendency). Among women co-residing with their mother-in-law, those with unintended pregnancies had higher odds of experiencing mental health issues compared to those with intended pregnancies (OR = 1.81, 95% CI = 0.98–3.34 for depression tendency; OR = 2.47, 95% CI = 1.40–4.38 for anxiety tendency; OR = 2.60, 95% CI = 1.17–5.74 for stress tendency), exceeding the levels observed among women not co-residing with their mother-in-law.

**Table 4:** Heterogeneity in the association between unintended pregnancy and mental health tendencies.

Subgroup	Independent variable	Depression tendency		Anxiety tendency		Stress tendency	
		OR (95% CI)	*p*-value	OR (95% CI)	*p*-value	OR (95% CI)	*p*-value
Women with different age							
<29	Unintended Pregnancy	1.36 (0.64, 2.87)	0.419	2.08 (1.08, 4.01)	0.028*	2.34 (0.95, 5.74)	0.063
≥29	Unintended Pregnancy	1.60 (0.77, 3.33)	0.213	2.18 (1.09, 4.34)	0.028*	2.45 (0.92, 6.51)	0.073
Women with different employment status							
Unemployed	Unintended Pregnancy	1.24 (0.72, 2.12)	0.435	2.05 (1.25, 3.35)	0.004**	2.20 (1.11, 4.34)	0.023*
Employed	Unintended Pregnancy	2.81 (0.34, 23.06)	0.336	1.84 (0.39, 8.69)	0.441	8.99 (0.33, 24.51)	0.191
Co-resident with mother-in-law							
No	Unintended Pregnancy	0.46 (0.15, 1.41)	0.176	1.15 (0.50, 2.68)	0.74	1.64 (0.44, 6.08)	0.462
Yes	Unintended Pregnancy	1.81 (0.98, 3.34)	0.057	2.47 (1.40, 4.38)	0.002**	2.60 (1.17, 5.74)	0.018*

*p < 0.05, **p < 0.01. Each subgroup represents separate regressions for depressive tendencies, anxiety tendencies, and stress tendencies. Only the odds ratio (OR), 95%CI and p-value of the independent variable for unintended pregnancy are presented, while the control variables are not included.

## 4 Discussion

Based on a sample collected from five prefectures and 10 counties, our study reveals that unintended pregnancies are highly prevalent in rural China, with a prevalence rate as high as 41.44%. This prevalence is comparable to some developing countries; for instance, the prevalence of unintended pregnancies among married women in Angola was reported to be 38.3% (39), while rates of approximately 40% were observed in Ethiopia, Jordan, and Nepal (40, 41). Unintended pregnancies are more common in developing countries due to inadequate sex education, limited contraceptive knowledge, and restricted access to reproductive health services which are typically constrained in developing countries (42, 43). Notably, this prevalence exceeds that found in developed countries and urban areas, where the unintended pregnancy rate is approximately 28% (44, 45). It is worth highlighting that our findings show no significant association between the prevalence of unintended pregnancies and women’s age or educational level. Consequently, women not only lack opportunities to acquire comprehensive knowledge about sex and contraception as they age but also face an absence of formal school education on these matters (9, 46–48). Therefore, additional interventions are required to mitigate its occurrence.

Our analysis found a significant association between unintended pregnancy and increased risk of anxiety and stress. Unintended pregnancy, being unwanted or mistimed, can profoundly impact maternal mental health through various mechanisms (49, 50). Firstly, their ability to handle the responsibilities of motherhood was not ready. They require knowledge acquisition on infant development and nurturing skills. Given the unintended nature of the pregnancy, they may not be adequately prepared for this role. Secondly, their time management is not ready. Unintended pregnancy can result in disruptions to their future plans, such as career and living arrangements (4). Thirdly, their financial preparedness may be not ready. Previous studies have demonstrated that raising an additional child leads to a significant increase in family expenditures by approximately 80–90% (51). Considering the unintended nature of the pregnancy, they may not be equipped to bear this additional financial burden (50, 52). Therefore, the lack of preparation for the arrival of a new baby may result in heightened mental health problems such as anxiety and stress. In addition, we found that when the good health status of maternal women is good and the household size is greater than 7, it has a positive impact on maternal mental health. Good maternal health reduces the risk of gestational diseases like diabetes and high blood pressure, and therefore less likely to suffer from mental health problems such as stress. Meanwhile, our data shows that in households with more than 7 members, maternal women own mothers more likely residing with them, which can provide more emotional support, family care, and communication opportunities.

The different impacts of unintended pregnancy on mental health in various characteristic groups. Firstly, we observed a consistent association between unintended pregnancy and maternal mental health across all age groups. This suggests that the need for additional support to cope with unintended pregnancy is not solely attributed to their young age. Secondly, unemployed pregnant women may experience a greater impact on their mental well-being from unintended pregnancy compared to their employed counterparts. This suggests that employment commitments may distract employed women from family life, making them more vulnerable to the disruptive effects of unintended pregnancy on their career plans. Thirdly, our findings indicated that pregnant women who co-reside with their mother-in-law experience a heightened impact of unintended pregnancy on maternal mental health. In China, the prevalent issue of intergenerational conflicts between mother-in-law and daughter-in-law often arises from disparities in concepts, ideas, and behaviors (53–55), particularly when facing unexpected pregnancies. Contrary to expectations, residing with the mother-in-law not only fails to alleviate their psychological distress but may even exacerbate the situation.

In summary, to our knowledge, this study is one of the limited research examining the association between unintended pregnancy and mental health in rural western China. The findings from this study serve as a representative and significant contribution to addressing the mental health concerns faced by women with unintended pregnancies. Our findings suggested a positive correlation between unintended pregnancies and an increased likelihood of mental health problems among pregnant women. The families often encounter barriers to accessing effective contraception due to financial constraints, limited healthcare resources, and inadequate support for maternal and child health services (56, 57). This finding underscores the importance of comprehensive reproductive health services, including access to contraception and mental health support. Addressing this issue requires reducing stigma, improving education, and providing robust support systems for unintended pregnancy women. Further research should explore the underlying mechanisms and potential interventions to mitigate the mental health impacts of unintended pregnancies.

Therefore, enhancing maternal health services emerges as a pivotal strategy to mitigate the repercussions associated with unintended pregnancies. Firstly, there is an urgent imperative to enhance efforts in disseminating comprehensive and accessible contraceptive knowledge. Relevant and age-appropriate sexual education programs, encompassing topics such as physical development, reproductive health, and contraceptive methods, should be incorporated into the formal school education. Additionally, comprehensive sex education and training should be made available to rural women in preparation for marriage. Secondly, the local public health service should prioritize maternal mental health issues by implementing comprehensive measures. These include providing specialized mental health training for local healthcare providers, implementing systematic screening programs for identifying maternal mental health problems, and recruiting additional experts to enhance the capacity of rural health bureaus. Thus, a robust and effective service system for addressing maternal mental health concerns can be established.

## 5 Limitation

However, it is imperative to acknowledge the limitations of this study. Firstly, our focus was solely on maternal mental well-being, neglecting other family members, particularly the mental well-being of their husbands. Secondly, the sample for our study was exclusively drawn from rural regions in western China. Therefore, caution must be exercised when extrapolating these findings. Thirdly, although this study was cross-sectional study design could not allow to assess causality between unintended pregnancy and maternal mental health. Fourthly, unintended pregnancy may be influenced by local culture, yet this study was unable to explore this potential factor and its role in explaining the correlation between unintended pregnancy and maternal mental health.

## 6 Conclusion

Our study demonstrated a positive correlation between unintended pregnancy and maternal health risks, suggesting that women who experience unintended pregnancies may not be adequately prepared for parenthood. Furthermore, these effects may be more pronounced in relation to employment status or family dynamics. Therefore, it is imperative to effectively disseminate contraceptive knowledge through education and training initiatives to mitigate potential negative consequences. Additionally, the local public health service requires comprehensive interventions to screen for and treat mental health issues among pregnant women residing in rural areas. Finally, future researches should be conducted on a larger scale to enhance sample representativeness, used longitudinal designs to assess causality, and incorporate considerations of local culture.

## Data Availability

The raw data supporting the conclusions of this article will be made available by the authors, without undue reservation.
